# Metastasis-Directed Radiation Therapy with Consolidative Intent for Oligometastatic Urothelial Carcinoma: A Systematic Review and Meta-Analysis

**DOI:** 10.3390/cancers14102373

**Published:** 2022-05-11

**Authors:** Nicola Longo, Giuseppe Celentano, Luigi Napolitano, Roberto La Rocca, Marco Capece, Gianluigi Califano, Claudia Collà Ruvolo, Francesco Mangiapia, Ferdinando Fusco, Simone Morra, Carmine Turco, Francesco Di Bello, Giovanni Maria Fusco, Luigi Cirillo, Crescenzo Cacciapuoti, Lorenzo Spirito, Armando Calogero, Antonello Sica, Caterina Sagnelli, Massimiliano Creta

**Affiliations:** 1 Department of Neurosciences, Science of Reproduction and Odontostomatology, University of Naples Federico II, 80131 Naples, Italy; nicola.longo@unina.it (N.L.); giuseppe.celentano2@unina.it (G.C.); roberto.larocca@unina.it (R.L.R.); marco.capece@unina.it (M.C.); gianluigi.califano@unina.it (G.C.); claudia.collaruvolo@unina.it (C.C.R.); francesco.mangiapia@unina.it (F.M.); simone.morra@unina.it (S.M.); carmine.turco2@unina.it (C.T.); francesco.dibello@unina.it (F.D.B.); giovanni.fusco@unina.it (G.M.F.); luigi.cirillo2@unina.it (L.C.); crescenzo.cacciapuoti@unina.it (C.C.); massimiliano.creta@unina.it (M.C.); 2 Department of Woman, Child and General, Specialized Surgery, Urology Unit, University of Campania “Luigi Vanvitelli”, 80131 Naples, Italy; ferdinando.fusco@unicampania.it (F.F.); lorenzo.spirito@unina.it (L.S.); 3 Department of Advanced Biomedical Sciences, University of Naples Federico II, Va Pansini, 5, 80131 Naples, Italy; armando.calogero2@unina.it; 4 Department of Precision Medicine, University of Campania “Luigi Vanvitelli”, 80131 Naples, Italy; antonello.sica@fastwebnet.it; 5 Department of Mental Health and Public Medicine, Section of Infectious Diseases, University of Campania “Luigi Vanvitelli”, Largo Madonna delle Grazie n. 1, 80138 Naples, Italy; caterina.sagnelli@unicampania.it

**Keywords:** bladder cancer, upper urinary transitional cell carcinoma, urothelial carcinoma, oligometastatic disease, radiotherapy

## Abstract

**Simple Summary:**

Patients with oligometastatic urothelial carcinoma represent a challenging subset of subjects to manage. Herein we summarized evidence about the role of metastasis-directed radiation therapy in this clinical setting. Available preliminary evidence supports the role of metastasis-directed radiation therapy as a safe and efficacious option as it has the potential to facilitate local disease control and overall survival. However, in the absence of data from high-quality trials, definitive recommendations cannot be provided, and patients should be counseled on an individual basis.

**Abstract:**

The management of patients with oligometastatic urothelial carcinoma (UC) represents an evolving field in uro-oncology, and the role of metastasis-directed therapies, including metastasectomy and metastasis-directed radiation therapy (MDRT), is gaining increasing attention. Herein, we summarize available evidence about the role of MDRT with consolidative intent in oligometastatic UC patients. A systematic review was performed in December 2021. Six studies involving 158 patients were identified. Most patients (*n* = 120, 90.2%) had a history of bladder cancer and the most frequent sites of metastases were lymph nodes (*n*= 61, 52.1%) followed by the lungs (*n* = 34, 29%). Overall, 144 metastases were treated with MDRT. Median follow-up ranged from 17.2 to 25 months. Local control rates ranged from 57% to 100%. Median Overall Survival (OS) ranged from 14.9 to 51.0 months and median progression-free survival ranged from 2.9 to 10.1 months. Rates of OS at one and two years ranged from 78.9% to 96% and from 26% to 63%, respectively. Treatment-related toxicity was recorded in few patients and in most cases a low-grade toxicity was evident. MDRT with consolidative intent represents a potential treatment option for selected patients with oligometastatic UC.

## 1. Introduction

Urothelial carcinoma (UC) represents the fourth and twelfth most common malignancy in men and women, respectively, with the bladder being the usual site of occurrence [1,2,3,4,5]. Despite radical therapies, a significant percentage of patients diagnosed with UC develop metastases during follow-up. In detail, metastatic disease is detected in about 50% of patients with bladder cancer undergoing radical cystectomy and most of these patients experience distant recurrence [1,2,3,6]. Moreover, 10 to 15% of patients are already metastatic at diagnosis [2]. Before the development of effective chemotherapy, patients with metastatic UC had a median survival rarely exceeding three to six months [1,2,3]. Although the treatment landscape of metastatic UC has been revolutionized in recent years with the advent of immune checkpoint inhibitors, available therapeutic options remain limited, and overall response rates remain suboptimal [1]. Metastatic UC patients represent a heterogeneous group of subjects. In 1995, Hellmann and Weichselbaum [7] hypothesized the existence of an intermediate clinical entity in the sequence of events leading to the progressive acquirement of metastatic ability from localized to metastatic disease: oligometastatic disease (OMD). OMD patients show distant relapse only in a limited number of regions and OMD is becoming more frequently identified thanks to the introduction of highly sensitive diagnostic modalities [8]. Although significant heterogeneity exists in the current OMD definitions in the literature, a recent ESTRO-ASTRO consensus document defined OMD as one to five metastatic lesions, a controlled primary tumor being optional, but where all metastatic sites must be safely treatable [9]. In recent years, the concept of OMD has been extended to UC patients, too [10]. Oligometastatic UC represents a rapidly evolving issue and the management of these patients in everyday clinical practice remains challenging due to the absence of clear recommendations from available guidelines. Metastasis-directed therapies (MDTs), such as metastasectomy and radiotherapy, have been used in the treatment of oligometastatic malignancies such as colorectal cancer with long-term benefits. The use of MDT has gained interest and popularity in the field of uro-oncology for the treatment of oligometastatic renal cell carcinoma and prostate cancer. Several reports, including both upper urinary tract transitional cell carcinoma and bladder cancer patients, support the hypothesis that resection of metastatic lesions could be safe and oncologically beneficial in selected patients with adequate life expectancy [3,11]. More recently, metastasis-directed radiation therapy (MDRT) with consolidative intent has emerged as a potential alternative to metastasectomy in UC patients with OMD. The aim of the present systematic review is to summarize available evidence about the role of metastasis-directed radiation therapy (MDRT) with consolidative intent in patients with oligometastatic UC.

## 2. Materials and Methods

The present analysis was conducted and reported according to the general guidelines recommended by the Primary Reporting Items for Systematic Reviews and Meta-Analyses (PRISMA) statement [12]. This protocol was registered in PROSPERO (CRD42021292657).

### 2.1. Literature Search

The search was performed in the Medline (US National Library of Medicine, Bethesda, MD, USA), Scopus (Elsevier, Amsterdam, The Netherlands), and Web of Science Core Collection (Thomson Reuters, Toronto, ON, Canada) databases up to January 2022. The following terms were combined to capture relevant publications: “oligometastatic” AND (“metastasis directed radiation therapy” OR “consolidative radiotherapy”) AND (“bladder cancer” OR “upper urinary tract transitional cell carcinoma” OR “urethra carcinoma” OR “urothelial carcinoma”). Reference lists in relevant articles and reviews were also screened for additional studies. Conference abstracts were also considered.

### 2.2. Selection Criteria, Data Collection, and Statistical Analysis

Two authors (N.L. and G.C.) reviewed the records separately and individually to select relevant publications, with any discrepancies resolved by a third author (L.N.). To assess eligibility for the systematic review, PICOS (participants, intervention, comparisons, outcomes, and study type) criteria were used [13]. PICOS criteria were set as follows: Participants—patients with oligometastatic UC; Intervention—MDRT with consolidative intent; Comparator—none; Outcome—response rate according to Response Evaluation Criteria in Solid Tumors (RECIST) criteria, Overall Response Rate (ORR), Local Control Rate (LCR), Overall Survival (OS), 1-year OS, 2-year OS, Progression-Free Survival (PFS), 1-year PFS, 2-year PFS, 3-year PFS, toxicity; Study types—conference abstracts, case reports, case series, retrospective and prospective studies. The following data were extracted from eligible studies: first author, year of publication, study design, sample size, patient age, patient gender, primary tumor site and stage, treatment for primary tumor, definition of OMD, OMD classification (synchronous, metachronous, oligoprogressive), performance status (PS), site, number and size of metastases, time to metastasis, MDRT type, overall number of treated lesions, number of lesions treated per patient, metastatic sites treated, metastases not treated, delivered radiation dose, dose fractionation, contemporary systemic treatments, previous systemic treatments, follow-up duration, response rate according to RECIST criteria, ORR, LCR, OS, 1-year OS percentage, 2-year OS percentage, PFS, 1-year PFS, 2-year PFS, 3-year PFS, and MDRT-related toxicity. The quality of included studies was assessed using the Jadad Score or the Methodological Index for Non-Randomized Studies (MINORS) for randomized and non-randomized studies, respectively [14,15]. Ethical approval and patient consent were not required for the present study. The meta-analysis was performed using ProMeta 3 software (Version 2.1) when two or more studies reported the same outcome under the same definition. The effect size (ES) was estimated using the event rate reported with a 95% confidence interval (CI). Heterogeneity among studies was evaluated using I^2^ statistics. A *p*-value < 0.05 was considered statistically significant. To calculate the pooled effect, a fixed effect model was applied. Egger’s linear regression test and Begg and Mazumdar’s rank correlation test were also used to evaluate the publication bias of studies included in the meta-analysis.

## 3. Results

**Figure 1:**
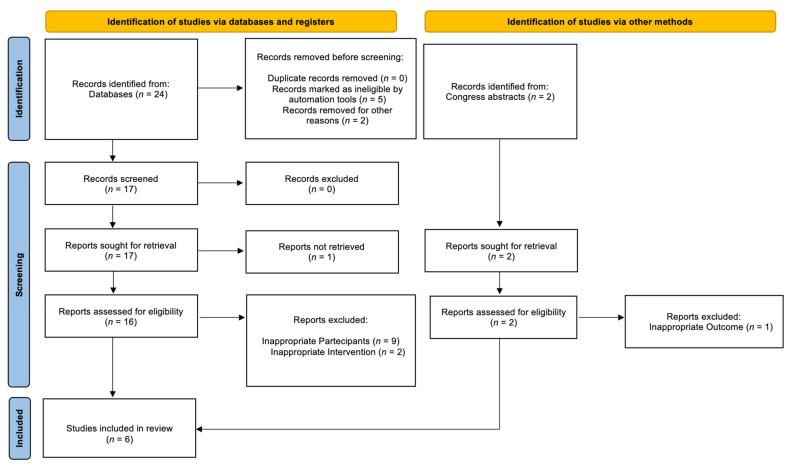
Flow diagram of the systematic review.

The search strategy generated a total of 26 results. The screening of the titles and abstracts determined 18 papers eligible for inclusion. Further assessment of eligibility, based on full-text study of the papers, led to the exclusion of 12 papers. Finally, 6 studies (1 conference abstract and 5 full papers) involving 158 patients were included in the final analysis (Figure 1) [16,17,18,19,20,21].

### 3.1. Patient Demographics and Tumor Characteristics

Overall, 6 retrospective studies were identified involving a total of 158 patients. Study characteristics, patient demographics, and tumor features are summarized in Table 1. Most of the patients included were male (*n* = 90, 56.9%), with a median age ranging from 63 to 71 years. The most frequent primary tumor site was the bladder (*n* = 120, 90.2 %). Radical surgery of the primary tumor was performed in 40 patients (39.6 %). The median time to metastasis was 15.6 months (range: 0–121.8) and the most common sites of metastases were lymph nodes (*n* = 61, 52.1%) followed by the lungs (*n* = 34, 29.0%). The type of OMD according to time of occurrence was reported in four studies. Most patients (59.0% to 100%) had metachronous oligometastatic disease (Table 1). The size of metastases was reported by only two authors [17,18]. In the study by Leonetti et al., the diameter of metastases ranged from 10 to 55 mm [17]. In the study by Augugliaro et al. [18], mean metastatic volume ranged from 3 to 88.7 cm^3^.

### 3.2. MDRT Protocols

MDRT details are summarized in Table 2. A total of 144 lesions were treated. Details about irradiates sites were provided by three studies. The most common irradiated sites were lymph nodes (*n* = 44). In two studies, MDRT was performed to treat all metastatic sites. MDRT was performed through Volumetric Modulated Arc Therapy (VMAT) in 67 patients (59.8%) and through CyberKnife (Accuracy Inc., Sunnyvale, CA, USA) in 15 patients (2.39%). The median delivered radiation dose ranged from 25 to 55 Gy. Dose fractionation was only reported by Augugliaro et al. (median: 5; range: 3–10) and by Franzese et al. (range: 1–10) [18,20]. Overall, 75 patients (47.4 %) received systemic therapy before MDRT, while 36 patients (22.7 %) received systemic therapy during MDRT. The median duration of follow up was 37.73 months (range: 3–91).

### 3.3. MDRT Outcomes

**Figure 2:**
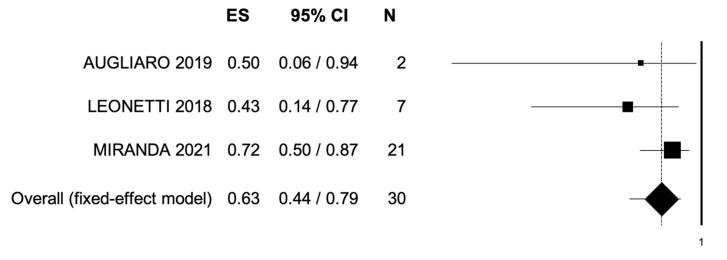
Forest plot showing the event rate for Overall Response Rate. ES, effect size; CI, confidence interval. (I^2^ = 0.00, *p* = 0.368.)

**Figure 3:**
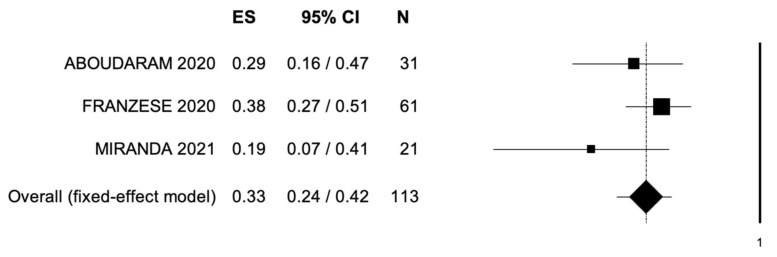
Forest plot showing the event rate for 2-year progression-free survival. ES, effect size; CI, confidence interval. (I^2^ = 25.39, *p* = 0.262.)

**Figure 4:**
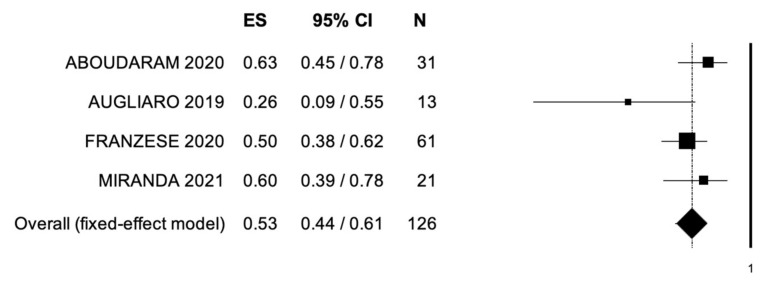
Forest plot showing the event rate for 2-year overall survival. ES, effect size; CI, confidence interval. (I^2^ = 42.91, *p* = 0.154.)

**Figure 5:**
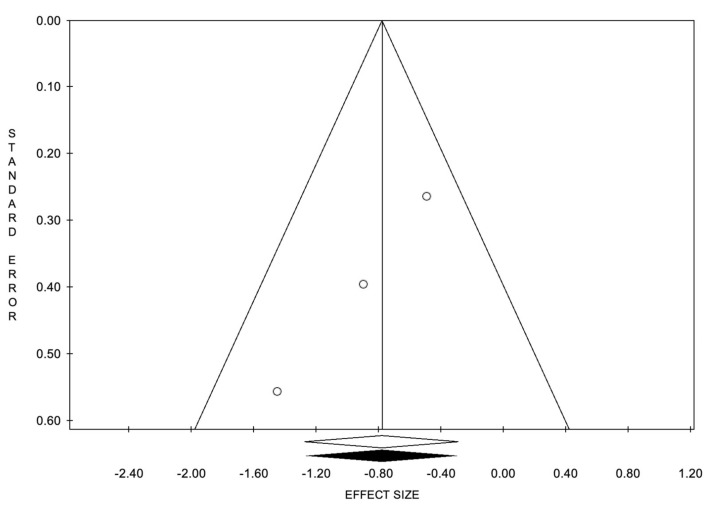
Funnel plots of the meta-analysis evaluating the event rate for 2-year progression-free survival. Egger’s linear regression (t= −28.93, *p* = 0.022) and Begg and Mazumdar’s rank correlation test (z = −1.57, *p* = 0.117).

**Figure 6:**
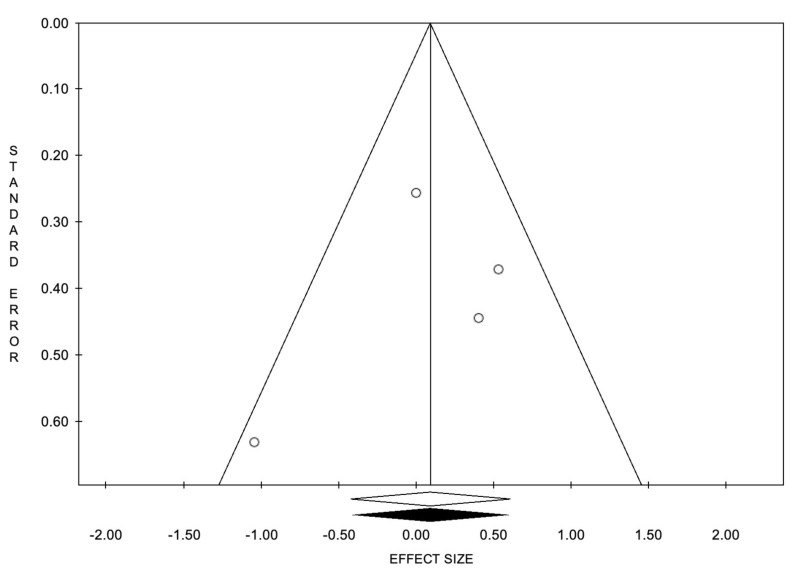
Funnel plots of the meta-analysis evaluating the event rate for 2-year overall survival. Egger’s linear regression (t= −0.47, *p* = 0.683) and Begg and Mazumdar’s rank correlation test (z = −0.68, *p* = 0.497).

**Figure 7:**
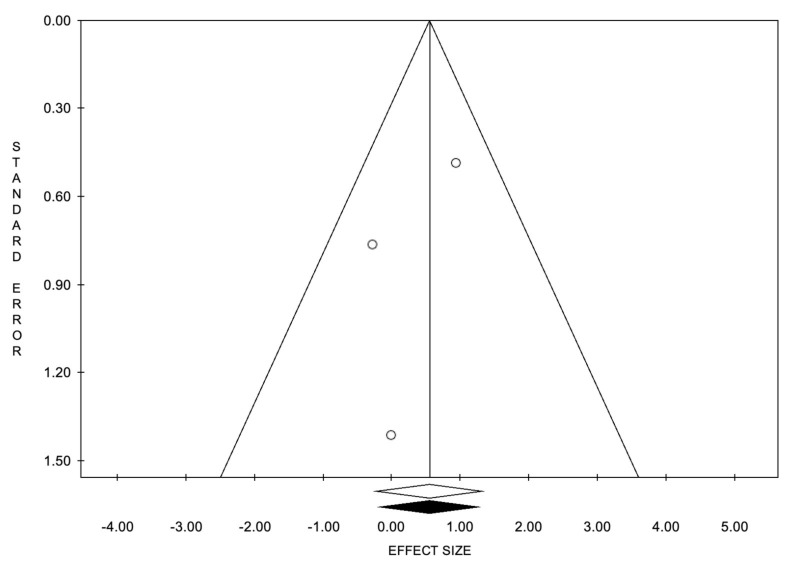
Funnel plots of the meta-analysis evaluating the event rate for Overall Response Rate. Egger’s linear regression (t = −0.98, *p* = 0.505) and Begg and Mazumdar’s rank correlation test (z = −0.52, *p* = 0.602).

The outcomes of MDRT are reported in Table 3. Local control was assessed through RECIST criteria in 56 patients (35.4%). CR, PR, and SD were observed in 14 (25.0%), 12 (21.4%), and 15 (26.7%) patients, respectively. PD was observed in 5 patients (8.9%). ORR and LCR were observed in a percentage of patients ranging from 38% to 57% and from 57% to 100%, respectively. Median OS was reported in 4 studies and ranged from 12.3 to 54.0 months. Rates of OS at 1- and 2-year follow-ups ranged from 78.9% to 96.0% and from 26.0% to 63.0%, respectively. A single study reported 3-year OS rates (43.3%). Median PFS was reported in 4 studies and was 20.8 months (range: 1.4–5.5). PFS rates at 1- and 2-year follow-ups ranged from 47.9% to 71.0% and from 19.0% to 38.1%, respectively. Pooled data from studies reporting ORR, 2-year PFS, and OS are reported in Figure 2, Figure 3 and Figure 4. Bias evaluation is reported in Figure 5, Figure 6 and Figure 7. Toxicity was evaluated in five studies. Grade 1 acute and late toxicities were reported in six and two patients, respectively. Grade ≥2 toxicity was reported in six patients.

## 4. Discussion

Recently, the overall perspective on metastatic disease has changed dramatically, due to both the availability of novel imaging modalities and the improvement of treatment options. With respect to metastatic UC patients, there have been two major advancements that have significantly modified their prognoses. On the one hand, immune checkpoint inhibitors have emerged as a promising alternative to conventional chemotherapy, with the advantage of improving objective response rates [20,21,22,23,24,25,26,27,28,29,30]. On the other hand, the concept of OMD, firstly introduced by Hellman et al. in 1995 as that of tumor states intermediate between purely localized and widely metastatic disease, has also been extended to UC, and patients with low burden of disease have been considered amenable to local treatment of metastatic disease [7,20]. Metastases represent potential sources of further metastatic spread. Therefore, the rationale behind MDT is that the eradication of a low number of metastases will hamper further metastatic dissemination, thus improving PFS and OS [31]. Typically, MDTs can be achieved by surgical metastasectomy or MDRT. Patient selection and treatment allocation represent a debated and complex topic when dealing with oligometastatic patients. Surgical metastasectomy has been incorporated into National Comprehensive Cancer Network guidelines as an option for selected oligometastatic UC patients with good response to systemic therapy [32]. However, due to the often extensive and difficult nature of the surgery, this procedure can be proposed only for highly selected patients with adequate performance and comorbidity status [32]. Traditionally, radiotherapy has been limited to disease palliation. In recent years, MDRT with consolidative intent has emerged as a more-attractive alternative to metastasectomy in oligometastatic patients thanks to its being less invasive [19]. The SABR-COMET and ORIOLE randomized trials demonstrated improved oncologic outcomes with MDRT in patients with several metastatic cancers [33,34].

Herein, we have summarized for the first-time available evidence about the role of MDRT with consolidative intent in patients with oligometastatic UC. Current evidence mainly derives from retrospective small series mainly involving oligometastatic bladder cancer patients with a history of radical surgery. Baseline patient characteristics deserve consideration as they reveal the tendency to select metastatic UC patients at good prognosis for treatment with MDRT. Most patients were young, had lymph node metastases, and good PS. These characteristics define a subgroup of patients with good prognoses. Indeed, a Karnofsky PS of ≤80% and presence of visceral metastases have been reported to represent independent prognostic factors of poor survival after treatment with MVAC, and these prognostic factors have also been validated for newer combination chemotherapy regimens [2,35]. Another relevant aspect is the timing of occurrence of OMD. In this context, OMD is commonly classified as synchronous or metachronous (often referred to as oligorecurrent) based on the time interval between primary cancer diagnosis and the development of OMD [9]. Metachronous OMD occurs when metastases are diagnosed 3–6 months from the diagnosis of the primary tumor. Most patients enrolled in the studies summarized in the present review had metachronous disease with a median time between diagnosis of the primary tumor to metastasis ranging from 12.9 to 2 months. The length of the disease-free interval has been reported as having a prognostic impact [9]. In patients with oligometastatic non-small cell lung cancer, metachronous metastases represent a significant predictor for improved OS [36].

Results from the present systematic review show LCRs (tumor volume equal to or less than the tumor volume at the start of radiotherapy) ranging from 72.0% to 100% and ORRs (complete or partial response) ranging from 38% to 57%. In comparison, in their recent systematic review assessing the outcomes of radiotherapy in oligometastatic prostate cancer patients, Rogowsky et al. reported LCRs ranging between 76 and 100% at 2 years [37].

Although response to therapy is commonly performed through computerized tomography, improvements in response evaluation are considered necessary and the use of radionuclides may represent a valid alternative [38].

Median OS ranges from 14.9 to 51.0 months with a median PFS ranging from 2.9 to 8.2 months. Of note, Muilwijk et al. found a median OS of 98.2 months in their subset of 22 oligometastatic UC patients undergoing metastasectomy [1]. In comparison, Rogowsky et al. reported PFS values ranging from 38 to 100% at 1 year and from 22–83% at 2 years and a median PFS ranging from 7 to 63 months [37]. Predictors of MDRT efficacy have been investigated only in a few cases. In the study by Miranda et al., the exploratory analysis of factors influencing OS among patients treated with MDRT with consolidative intent showed improved OS in patients treated with concurrent systemic therapy during MDRT (51 vs. 12 months, *p* = 0.01) and no difference with respect to ECOG performance status, number of metastatic sites, radiation modality, and radiologic response in the target lesion [19]. Interestingly, the majority (67%) of patients in the study by Miranda et al. received concurrent treatment with immuno-oncology agents. In this context, preliminary evidence suggests the existence of a potential synergistic effect deriving from the combination of immuno-oncology agents and MDRT [35]. Recent evidence suggests that the tumor immune microenvironment can be stimulated by radiation. Indeed, radiation can lead to the activation of adaptive and innate immune response, with activation of cytotoxic T cells following the release of tumor antigens caused by cell death [39]. Other prognostic variables associated with better outcomes include higher dose of SBRT, lower number of metastases, and lower number of lines of systemic therapy before SBRT. Of note, the available evidence demonstrates that MDR carries minimal morbidity, with only a minority of patients experiencing toxicity. Although this finding is in line with published studies on MDRT, data are still poor and suboptimal and further studies are needed to confirm long-term safety [33,34]. Evidence synthesized in the present review indicates the limitations of the current literature regarding the role of MDRT in oligometatstic UC patients and provides a basis for further investigations. Major limits of the available studies include retrospective, single-institution, and often uncontrolled designs, small sample sizes, and short follow-up periods. Moreover, series are heterogeneous in terms of patient characteristics, definitions of oligometastatic states, treatment protocols, and outcomes assessed. Finally, MDRT has been carried out at different time-points along the natural history of the disease and details about the type of chemotherapy performed before, during, or after MDRT are often unavailable. Future research efforts should include rigorous definitions of diagnostic modalities and criteria for the definition of OMD in UC patients. Despite promising preliminary results, the role of fluorodeoxyglucose-positron emission tomography-computed tomography deserves further investigation [31,40]. Molecular biomarkers such ad miRNAs and exosomes that have been reported to be involved in the regulation of the metastatic cascade and in the switch from oligometastatic to polymetastatic states may represent potential alternatives [41]. Moreover, predictors of response to MDRT should be carefully investigated to adequately select patients as candidates for this treatment strategy. In this context, a phase II, multicenter, randomized open-label, and comparative study designed to evaluate whether local consolidative radiotherapy plus standard of care can improves OS as compared to standard of care in patients with limited metastatic urothelial bladder cancer and without progression following the initial phase of first-line systemic therapy is ongoing (ClinicalTrials.gov Identifier: NCT04428554).

## 5. Conclusions

In conclusion, available evidence supports the role of MDRT as a safe and efficacious option in treating selected oligometastatic UC patients. It has the potential to facilitate local disease control and overall survival. Nonetheless, in the absence of data from randomized controlled trials, patients should be evaluated on an individual basis and the decision to perform MDRT with consolidative intent should be made in a joint decision-making process with the patient.

## Figures and Tables

**Table 1:** Study characteristics and clinico-pathological features of the patients enrolled.

Author	MINORS	Sample Size (*n*)	Age at MDRT, Years, Median (Range)	M: F	Primary Tumor			OMD Criteria	Time to MTX, Mo, Median (Range)	OMD Classification (*n*, %)	PS	MTX Site (*n*)
					Site (*n*)	Stage (*n*)	Treatment Type (*n*)					
Leonetti [17]	10	7	67 (47–79)	6:1	B (5) P or Ur (2)	pT3N3cM0 (1) pT2N3cM1 (1) pT2N0cM0 (1) pT1cN0M0 (1) pT3N1cM0 (1) pT2cN0M0 (1) pT3cN0M0 (1)	Radical surgery (7)	≤3 MTX and no liver MTX	12.9 (0–33.6)	SO (1, 14.2) MO (6, 85.7)	n.a.	Ln (14)
Augugliaro [18]	9	13	68 (50–80)	n.a.	B (13)	n.a.	RC (12)	≤5 MTX	23.0 (8–105)	MO (13, 100)	90 (80–90) *	Ln (18) Lung (1) Pelvic recurrence (1) Pelvic bone (1)
Abe [16]	8	25	64 (45–79)	18:7	n.a.	n.a.	n.a.	MTX in a single organ with a small number of MTX	n.a.	n.a.	ECOG 0 (21) ECOG 1 (3) ECOG 2 (0) ECOG 3 (0) n.a. (1)	n.a.
Aboudaram [21]	-	31	n.a.	n.a.	B (31)	n.a.	n.a.	≤5 residual MTX and no disease progression following chemotherapy	n.a.	n.a.	n.a.	n.a.
Franzese [20]	10	61	71 (48.2–86.2)	51:10	B (50) P (7) Ur (3) U (1)	n.a.	Local control (61)	≤5 MTX and maximum diameter ≤5 cm	14.5 (0–121.8)	SO (3, 4.9) MO (36, 59.0) OP (22, 36.0)	n.a.	Lung (33) Ln (29) Liver (7) Bone (5) Adrenal (4) Other (4)
Miranda [19]	8	21	63 (n.a.)	15:6	B (21)	T2 (n.a.) T3 (n.a.)	RC (21)	≤4 MTX	12.1 (6.8–36) ^#^	SO (3) *^#^* MO (49)*^#^*	ECOG 0–1 (17) ECOG 2–5 (4)	n.a.

B: Bladder; ECOG: Eastern Cooperative Oncology Group; Ln: Lymph nodes; MO: Metachronous oligometastatic; n.a.: not available; P: Pelvis; PS: Performance Status; RC: Radical Cystectomy; Ur: Ureter; U: Urethra; *: Karnofsky Performance Status, median (range); ^#^: values calculated for the overall study population.

**Table 2:** MDRT details.

Author	MDRT Technique (*N*)	Overall Number of Treated Lesions (*n*)	Treated Lesions per Patient *n* (%)	Sites Treated Site (*n*)	Sites Not Treated, *n* (%)	Delivered Dose, Gy, Median (Range)	Systemic Therapy before MDRT Type (*n*)	Systemic Therapy during MDRT Type (*n*)	Fu, Mo, Median (Range)
Leonetti [17]	SBRT (14)	14	1 lesion: 2 (28.5) >1 lesions: 5 (71.4)	All sites	0	32 (25–40)	n.a. (2)	n.a. (1)	n.a.
Augugliaro [18]	IMRT (n.a.) CyberKnife (n.a.)	21	1 lesion: 8 (61.5) >1 lesions: 5 (38.4)	All sites	0	25 (20–36)	GC (7)	n.a.	25 (3–43)
Abe [16]	n.a.	27	1 lesion: 20 (80.0) >1 lesions: 5 (20.0)	Ln (12) Lung (1) Bone (5) Liver (5) Local recurrence (4) Bladder (3) Other sites (1)	4 (16.0)	55 (30–69)	(n.a.) (6)	n.a. (5)	n.a.
Aboudaram [21]	n.a.	n.a.	n.a.	n.a.	a.a.	>50	31 (100)	n.a.	71 (n.a.)
Franzese [20]	Cyberknife (15) VMAT (67)	82	1 lesion: 37 (60.7) >1 lesions: 24 (39.2)	n.a.	24 (39.3)	45 (18–70)	n.a. (29)	GC or carboplatin + gemcitabine, or vinflunine (14)	17.2 (3–91)
Miranda [19]	SBRT (16) Non-SBRT (5)	n.a.	1 lesion: 9 (43) >1 lesions: 12 (57.0)	n.a.	0	n.a.	n.a.	n.a. (16)	n.a.

Fu: Follow-up; GC: Gemcitabine + Cisplatin; IMRT: Intensity-Modutated Radiation Therapy; MDRT: Metastasis-Directed Radio Therapy; n.a.: not available; SBRT: Stereotaxic Body Radiation Therapy; VMAT: Volumetric Modulated Arc Therapy.

**Table 3:** MDRT outcomes.

Author	Response Rate ** Type, *n* (%)	ORR (%)	LCR (%)	OS, Mo, Median (95% CI)	1-Year OS (%)	2-Year OS (%)	3-Year OS (%)	PFS, Mo, Median (95% CI)	1-Year PFS (%)	2-Year PFS (%)	Systemic Therapy after MDRT Type (*n*)	Prognostic Variables	Toxicity Grade (*n*)
Leonetti [17]	PR, 6 (43.0) SD, 8 (57.0)	43.0	100	14.9 (12.3–17.5)	n.a.	n.a.	n.a.	2.9 (2.6–3.1)	n.a.	n.a.	n.a.	Higher dose of SBRT associated with higher lesion progression-free interval	0
Augugliaro [18]	CR, 11 (52.0) PR, 1 (5.0) SD, 0 (0) LPE, 8 (38.0) NE, 1 (5.0)	57.0	57.0	n.a.	n.a.	26.0	n.a.	5.8 (n.a.)	n.a.	n.a.	GC (1)	n.a.	Grade 1 (Acute) (1)
Abe [16]	n.a.	n.a.	n.a.	29.0 (17–54)	n.a.	n.a.	43.3	n.a.	n.a.	n.a.		n.a.	n.a.
Aboudaram [21]	n.a.	n.a.	n.a.	n.a.	96.0	63.0	n.a.	n.a.	71.0	29.0		n.a.	Grade ≥ 3 (0)
Franzese [20]	n.a.	n.a.	88.9	25.6 (n.a.)	78.9	50.7	n.a.	10.1 (n.a.)	47.9	38.1	n.a.	Lines of systemic therapy before SBRT associated with inferior local control. Higher number of metastases associated with inferior PFS Total delivered dose associated with OS	Grade 1 (Acute) (5) Grade 1 (Late) (2)
Miranda [19]	CR, 3 (14.0) PR, 5 (24.0) SD, 7 (34.0) PD, 5 (24.0) n.a., 1 (4.8)	38.0	72.0	51.0 (n.a.)	n.a.	60.0	n.a.	8.2 (1.4–5.5)	n.a.	19.0	n.a.	Concurrent systemic therapy during MDRT associated with improved OS	CTCAE Grade ≥ 2 (5) CTCAE Grade ≥ 3 (1)

CSS: Cancer-Specific Survival; CR: Complete Response; GC: Gemcitabine + Cisplatin; LCR: Local Control Rate; LPD: Local Progression Disease; MDRT: Metastasis-Directed Radiation Therapy; NE: not evaluable; n.a.: not available; OS: Overall Survival; ORR: Overall Response Rate; PD: Progressive Disease; PFS: Progression-Free Survival; PR: Partial Response; SCTCAE: Common Terminology Criteria for Adverse Events; SD: Stable Disease; **: According to RECIST (Response Evaluation Criteria in Solid Tumors) criteria.

## Data Availability

Not applicable.
